# An assessment of bovine herpes virus 4 as a causative agent in abortions and neonatal death

**DOI:** 10.4102/ojvr.v87i1.1761

**Published:** 2020-02-06

**Authors:** Seval B. Dağalp, Ali R. Babaoglu, Firat Doğan, Touraj A. Farzani, Feray Alkan

**Affiliations:** 1 Department of Virology, Faculty of Veterinary Medicine, Ankara University of Veterinary Medicine, Ankara, Turkey; 2 Department of Virology, Faculty of Veterinary Medicine, YüzüncüYil University, Van, Turkey; 3 Department of Virology, Faculty of Veterinary Medicine, Mustafa Kemal University, Hatay, Turkey

**Keywords:** abortion, BoHV-4, cow, co-infection, viruses

## Abstract

Numerous viruses, including bovine viral diarrhoea virus (BVDV), bovine herpes virus 1 (BoHV-1) and bovine herpes virus 4 (BoHV-4), and other pathogens are the most common causes of reproductive disorders and are responsible for huge economic losses in livestock production. This study investigates the aetiological role of BoHV-4 in fertility problems such as abortions, stillbirth and birth with unviable calves. Retrospective samples from 38 animals, including 17 aborting cows, 17 aborted foetuses, three stillborn calves and one unviable newborn calf were analysed. The BoHV-4 genome was detected in 25 (65.7%) animals by polymerase chain reaction. In 14 of these infected animals, we detected co-infection with BVDV, while the co-presence of BoHV-1 was also detected in one animal. In addition to the high prevalence of BoHV-4 genome in materials related to fertility problems, isolation of BoHV-4 from the brain of one stillborn calf indicated a causal link between BoHV-4 and fertility problems, such as abortion, stillbirths or birth with unviable calves.

## Introduction

Previous studies have identified several viruses associated with reproductive disorders in cattle, such as abortion, neonatal death and births with congenital abnormalities. In such cases, bovine viral diarrhoea virus (BVDV) and bovine herpes virus 1 (BoHV-1) (Czaplicki & Thiry 1998; Kirkbride 1992; McKercher & Wada 1964; Miller & Van der Maaten 1987; Murray 1990; Schiefer 1974; Wellemans, Van Opdenbosch & Mammerickx 1986) are often detected along with bovine herpes virus 4 (BoHV-4). The detection of BoHV-4 even in healthy cattle (Czaplicki & Thiry 1998; Kirkbride 1992; Monge et al. 2006) makes the investigation of the epidemiology and pathogenetic mechanisms of this infection an interesting subject for researchers.

Bovine herpesvirus 4 (BoHV-4) is a member of the *Rhadinovirus* genus of the *Gamma herpes virinae* sub-family (ICTV 2017). Antigenically, closely related strains of BoHV-4 with similar restriction patterns form two main groups, namely, genotype 1 and genotype 2, which possibly correspond to the Movar-like and DN 599-like reference strains, respectively. Genotype 3 has also been reported recently (Verna et al. 2012). As with other herpes viruses, animals experimentally infected with BoHV-4 develop a latent infection, followed by virus reactivation and re-excretion after dexamethasone treatment and stress factors such as calving (Dubuisson et al. 1989). This virus naturally infects blood mononuclear cells, such as macrophages and monocytes, and displays a specific tropism towards vascular endothelia, mammary tissue, endometrium and foetal tissues. Clinical manifestations associated with BoHV-4 in cattle include postpartum metritis (Monge et al. 2006), abortion (Czaplicki & Thiry 1998; Kirkbride 1992; Schiefer 1974; Wellemans et al. 1986), mastitis (Izumi et al. 2006; Miyano et al. 2004; Wellenberg et al. 2000), pneumonia, keratoconjunctivitis, encephalitis and diarrhoea in both natural and experimental infections (Goyal & Naeem 1992; Thiry et al. 1990). Although most BoHV-4 isolates are considered mild pathogens or completely apathogenic for cattle, BoHV-4 antibodies have higher prevalence in abortion cases than in clinically asymptomatic cattle (AltamIranda et al. 2015; Naeem, Goyal & Werdin 1989).

In Turkey, numerous studies have investigated BoHV-1 and BVDV as aetiological agents of fertility problems, such as abortion, and have conducted serological screening of antibodies against these agents (Alkan & Burgu 1993; Alkan et al. 2005, 2009, 2018; Bilge-Dagalp et al. 2008; Oğuzoğlu et al. 2012; Tan et al. 2006). There have also been virological and serological studies on BoHV-4 infection. These studies show that BoHV-4 infection is very common and is increasing significantly in cattle herds in Turkey (Aslan, Azkur & Gazyagc 2015; Bilge-Dagalp et al. 2007, 2008, 2011; Dağalp et al. 2010; Tuncer-Göktuna et al. 2016; Yildirim et al. 2011). However, there is no published data about the detection of BoHV-4 in samples from aborted foetuses and stillborn or unviable calves.

The present study investigates the presence of BoHV-4 genome in samples from aborting cows, aborted foetuses, stillborn calves and unviable calves to investigate the interaction between BoHV-4 and these fertility problems. It also investigated whether there is any significant association between co-infection of BoHV-4 and two other abortifacient pathogens, BVDV and BoHV-1.

## Materials and methods

### Sampled animals

We used retrospective samples from 38 animals submitted to our laboratory for routine diagnosis, including 17 aborted foetuses, four stillbirths or unviable calves and 17 aborting cows from four different cattle herds (totalling 500–700 animals). In total, the 26 samples from 17 cows comprised leukocytes (*n* = 15), vaginal swabs (*n* = 9) and placental materials (*n* = 2). Other samples comprised various tissues from foetuses aborted at seventh to eighth month of gestation and from stillborn or unviable calves (Table 1). To increase the possibility of detecting viruses, each sample from the same animal was tested for BoHV-4 by polymerase chain reaction (PCR) assay along with two other frequently detected pathogens in Turkey, namely, Bovine Herpes Virus 1 (BoHV-1) and BVDV.

**TABLE 1:** Distribution of materials and animals tested and polymerase chain reaction results.

Herd No.	Animal No.	Animals sampled	Samples tested	PCR results	
				BoHV-4	Other pathogens
I	1	Aborted foetus	Lung, brain, liver	Lung, brain	BVDV
	2	Stillbirth calf	Brain, spleen, lung, liver	Brain	BVDV
	3–4	Stillbirth calf	Spleen, lung, liver, brain	-	-
	5–7	Aborted foetus	Spleen, brain	-	BVDV
	8	Aborted foetus	Spleen, liver, brain, lung	-	BVDV
	9	Aborted foetus	Spleen, liver, brain, lung	Brain, lung	BVDV
	10	Aborted foetus	Brain, kidney, spleen, liver, heart	Brain	BVDV
	11	Aborted foetus	Liver, lung, brain, spleen	Liver, lung	-
	12	Aborted foetus	Spleen, liver, brain, lung, kidney	-	BVDV
	13	Aborted foetus	Brain, liver, kidney	Brain, liver, kidney	BVDV
	14	Aborted foetus	Brain, liver, kidney	Brain, liver, kidney	-
	15–16	Aborting cows	Leukocyte	-	-
	17	Aborting cows	Leukocyte	Leukocyte	-
	18	Aborting cows	Leukocyte	Leukocyte	BoHV-1
	19–26	Aborting cows	Vaginal swab, leukocyte	Vaginal swab	BVDV
	27	Aborting cows	Vaginal swab, leukocyte	Vaginal swab	-
	28	Aborting cows	Placental material	-	-
II	29–30	Aborted foetus	Spleen, liver, brain, lung	-	
	31	Aborted foetus	Liver, lung, spleen, brain	Brain	-
	32	Aborted foetus	Liver, leukocyte, lung, spleen, brain	Leukocyte	BVDV
	33	Aborted foetus	Brain, lung, liver, spleen	Spleen, liver, brain, lung	-
	34	Aborted foetus	Spleen, liver, brain, lung, kidney	Spleen, liver	-
	35	Aborting cows	Leukocyte	Leukocyte	-
III	36	Calf birth with unviable	Spleen, liver, brain, lung, kidney	-	-
	37	Aborting cows	Leukocyte	Leukocyte	-
IV	38	Aborting cows	Placental material	Placental material	-
**Total**		**30/38 (78.9%)**	**-**	**25/38 (65.7%)**	**19/38 (50.9%) for BVDV** **1/38 (2.6%) for BoHV-1**

PCR, polymerase chain reaction; BVDV, bovine viral diarrhoea virus; BoHV-4, bovine herpes virus 4; BoHV-1, bovine herpes virus 1.

### Viral nucleic acid extraction and polymerase chain reaction

Extraction of deoxyribonucleic acid (DNA) for all samples was carried out according to Sambrook, Fritsch and Maniatis (1989). Total RNA was extracted using a high pure viral nucleic acid kit (Roche, Germany) according to the manufacturer’s recommendations. Complementary DNA (cDNA) synthesis was performed using the First Strand cDNA Synthesis Kit (Thermo Scientific, United States) according to the manufacturer’s protocol.

The primer sets specific for *gB* of BoHV-4 F: (*5’-CCCTTCTTTACCACCACCTACA-3’*) and R: (*5’-TGCCATAGCAGAGAAACAATGA-3’*) (Goltz et al. 2004), *gC* of BoHV-1 F: (*5’-CTGCTGTTCGTAGCCCACAACG-3*’) and R: (*5’-TGTGACTTGGTGCCCATGTCGC-3’*) (Van Engelenburg et al. 1993) and *5’-UTR* of BVDV F: (*5’-ATG CCC WTA GTA GGA CTA GCA-3’*) and R: (*5’- TCA ACT CCA TGT GCC ATG TAC -3’*) (Vilcek et al. 1997) were used in the PCR techniques that are described elsewhere with some minor modifications (Wellenberg et al. 2001).

Specific PCR products (615 base pair (bp), 397 bp and 288 bp of BoHV-4, BoHV-1 and BVDV, respectively) were visualised in transilluminator after electrophoresis in 1% agarose gel containing 10-mg/mL ethidium bromide.

### Ethical considerations

This study was performed within the Scientific Research Projects of Ankara University (Project No.: 10B3338005).

## Results

The PCR results are shown in Table 1, including the description of animals and related materials. Out of 38 animals, the BoHV-4 genome was detected in 25 (65.7%) animals, with 14 (36.8%) and one (2.6%) co-infection rates for BVDV and BoHV-1, respectively. Moreover, out of 38 animals, 19 (50.9%) animals were found to be positive for BVDV and one (2.6%) animal was found to be positive for BoHV-1 (Table 1). The estimated rate of BoHV-4 positivity was 76.1% (16/21) for aborted foetuses and stillborn or unviable calves and 82.5% (14/17) for cattle (Table 2). In total, 78.9% (30/38) of the sampled animals were positive for at least one of the tested viruses. The data showed that almost all tissues from an aborted foetus with BoHV-4 were found positive (Table 1).

**TABLE 2:** Results according to sampled herds and clinical cases.

Animals (*n* = 38)	Herds	Tested animals	BoHV-4	Co-infections		BVDV	Total	
				BoHV-4 + BVDV	BoHV-4 + BoHV-1		*n*	%
Aborting cow (*n* = 17)	I	14	2	8	1	-	-	-
	II	1	1	-	-	-	-	-
	III	1	1	-	-	-	-	-
	IV	1	1	-	-	-	-	-
	Subtotal	17	5	8	1	-	14	82.5
				9				
Foetus and others (*n* = 21)	I	14	2	5	-	5	-	-
	II	6	3	1	-	-	-	-
	III	1	-	-	-	-	-	-
	Subtotal	21	5	6	-	5	16	76.1
				6				
**Total (*n* = 38)**			**-**	**14**	**1**	**-**	**-**	**-**
***n***			**10**	**15**		**5**	**30**	**-**
**%**			**47.6**	**57.0**		**23.8**	**-**	**78.9**

BVDV, bovine viral diarrhoea virus; BoHV-4, bovine herpes virus 4; BoHV-1, bovine herpes virus 1.

### Discussion

Bovine herpes virus 4 is a co-factor in cattle abortion cases along with other pathogens, including viruses, bacteria and protozoans. They also play a clear role in certain other reproductive disorders such as endometritis (Chastant-Maillard 2015; Cvetojević et al. 2016; Frazier et al. 2002; Reed, Langpap & Bergeland 1979). Researchers have, therefore, focussed on detecting BoHV-4 in semen, uterine and blood samples from aborting cows in herds with postpartum metritis (Cvetojević et al. 2016; Dağalp et al. 2012; Nikolin et al. 2007; Nikolin, Vesna & Radosavljević 2008). However, the connection between BoHV-4 and abortions remains unclear because the virus has been isolated from both diseased and healthy cattle (Czaplicki & Thiry 1998; Deim, Szeredi & Egyed 2007; Leboeuf 2013).

Our results (Tables 1 and 2) show that BoHV-4 infection has a very high prevalence in the samples obtained from Herds I and II. Because very few animals were sampled from Herds III and IV, we could not analyse the prevalence of BoHV-4 infection. However, our previous results and knowledge from veterinarians suggest that BoHV-4 infection is also increasing significantly in these herds, resulting in an increase in economic losses (Bilge-Dagalp 2007, 2008, 2011; Dağalp et al. 2012).

The pathogenesis of BoHV-4 infection has been questioned because of the detection of BoHV-4 from both healthy individuals and cattle with a wide variety of clinical signs (Monge et al. 2006; Wellenberg et al. 2000). In our study, out of 38 animals tested by PCR targeting the *gB* gene region of BoHV-4, 25 animals, including aborting cows (*n* = 14) and aborted, stillborn and unviable calves (*n* = 11), tested positive for the virus. Because of the use of retrospective samples, different materials (blood, vaginal and placental samples) could be tested for aborting cows. However, it is not yet possible to say which of these materials should be used to diagnose BoHV-4 infection, although vaginal specimens appear superior to blood samples (Table 1). Similarly, several tissue samples from an aborted foetus gave positive results for BoHV-4, which indicate that BoHV-4 is transferred to the foetus transplacentally. In addition, isolation of BoHV-4 from a brain sample of a stillborn calf (animal No. 2 in Table 1) in another study (unpublished data) is very important for showing transplacental transfer and the virus’ causative role in fertility problems. In short, our study has substantially determined a link between fertility problems such as abortion and stillbirths and BoHV-4 because we detected the virus in various foetal tissue samples, while the other studied pathogens, especially BVDV, were absent in most of the aborted foetuses (Tables 1 and 2). Verna et al. (2012) likewise concluded that the detection of BoHV-4 as a sole agent offers indirect evidence of the virus’ involvement in bovine abortion.

Several reports indicate that BoHV-4 has an immunosuppressive effect (Egyed 2000; Egyed et al. 1996) and contributes to disease development by stimulating inflammatory reactions (Donofrio et al. 2005). Szenci et al. (2016) reported that local multiplication of *Histophilus somni* may produce certain chemicals (PGE2) that reactivate latent BoHV-4 from some local macrophages, while multiplication of the reactivated virus decreases the efficiency of local macrophage functions (opsonisation, antigen presentation and killing activity), which further promotes local bacteria multiplication. For this reason, we also tested all our samples for BVDV and BoHV-1, which are very common infections in Turkey, as well as for BoHV-4. The rate of detection of BVDV was as high as for BoHV-4 in Herd I, which had an acceptable sample size of aborting cows and foetuses for this assessment (Table 2). One of the six aborted foetuses was also co-infected with BoHV-4 and BVDV. However, it is not possible to conclude whether this signifies an interaction between BVDV and BoHV-4 infection in these cases, although BoHV-4 has been identified as an important factor affecting the immune system. This could explain the high rates of these infections in Herds I and II. Based on our knowledge, few studies have investigated clinical cases, including abortions and the effects of BoHV-4 along or together with these other viruses. Yilmaz, Coskun and Sahin (2016) had reported that 66.66% of aborted calves (8/12) were positive for BVDV, although BoHV-1 and BoHV-4 were not detected in the sampled animals. Similarly, another study (Tuncer-Göktuna et al. 2016) using PCR analysis for investigating the role of herpes viruses and pesti-viruses in cases of ruminant abortion between 2007 and 2015 in western Turkey did not find BoHV-4 in any of the tested samples from 60 aborted foetuses, while two and 31 calves aborted analysed using ELISA were found positive for BoHV-1 and BVDV antigens, respectively. However, some other studies have reported the presence of BoHV-4 and its co-infection with other pathogens (Cvetojević et al. 2016; Frazier et al. 2002; Reed et al. 1979). Cvetojević et al. (2016) detected BoHV-4 in 21% of examined samples (21/100), co-infection of BoHV-4 with BVDV in two samples and one sample infected with Neospora caninum. Otherwise, several studies have proposed that BoHV-4 is a secondary agent, often associated with secondary bacterial infections (Chastant-Maillard 2015; Donofrio et al. 2008; Klamminger et al. 2017; Jacca et al. 2014; Nak et al. 2011) and also fungi or other viruses (Drolet, Werdin & Goyal 1986; Fabian et al. 2008; Frazier et al. 2002). Gagnon et al. (2017) suggested that because BoHV-4 is a frequent risk or a secondary factor in cattle infections, higher BoHV-4 seroprevalence in cattle with respiratory or reproductive diseases could be expected. Thus, our data detecting two viral pathogens in the same aborted foetus, stillborn calves and aborting cows (Tables 1 and 2) are not surprising. Unfortunately, we were unable to test both foetuses and their mothers. Therefore, more detailed investigations are needed to establish a clear link between BoHV-4 infection and abortion as well as the potential interaction of BoHV-4 with other abortifacient pathogens in the same sample.

In this study, there were two main limiting factors. Firstly, it did not test for the presence of any additional microorganisms (bacteria, etc.). Secondly, it did not include serological results to show the positivity rate for BoHV-4 or the other studied viruses in aborting cows and/or healthy cattle in these herds.

In conclusion, we consider that BoHV-4 alone or in conjunction with other pathogens, especially BVDV, contributed to the development of the reproductive disorders reported in this study, and possibly to others as well. Further studies using epidemiological data from BoHV-4 infections and molecular characterisation of field BoHV-4 in different clinical cases are needed to understand their pathogenesis and responsibility for economic losses affecting cattle husbandry in Turkey.
